# Supplementary material for: Motivations, perceived risk, and tampering of XTAMPZA ER and abuse-deterrent opioid drugs
Source: PLoS One. 2025 Oct 8;20(10):e0332574. doi: 10.1371/journal.pone.0332574 (PMC12507222; doi:10.1371/journal.pone.0332574)
Supplement: S1 Table — Product Listing of Drug Comparator Groups. (DOCX) [file pone.0332574.s001.docx]

**Supplemental Table 1**: Product Listing of Drug Comparator Groups

| Comparator Drug Group | Included Drugs/Substances |
| --- | --- |
| Other ADF ER oxycodone Or hydrocodone products | Oxycontin® pill |
|  | Hysingla® ER pill |
| non-ADF oxycodone or hydrocodone products | Generic extended release tablet containing only hydrocodone |
|  | Generic extended release pill containing only oxycodone |
| ir oxycodone products | Immediate release pill containing only oxycodone (*Oxaydo®, Oxecta®, Roxicodone®, or generics*) |
