# Supplementary material for: Motivations, perceived risk, and tampering of XTAMPZA ER and abuse-deterrent opioid drugs
Source: PLoS One. 2025 Oct 8;20(10):e0332574. doi: 10.1371/journal.pone.0332574 (PMC12507222; doi:10.1371/journal.pone.0332574)
Supplement: S1 File — (DOCX) [file pone.0332574.s002.docx]

We are interested in whether you have used the prescription medications below **in a way not directed by your healthcare provider in the last 12 months**, even if just once or just a little. Examples include:

 You used it without a prescription or with someone else’s prescription

 You used it in greater amounts, more often, or for longer than directed

 You used it for the experience or feeling it caused

 You changed it before use by crushing, chewing, dissolving, or heating it

Have you used the pain medication below **in the last 12 months** in a way not directed by your healthcare provider?

| Yes |
| --- |
| No |

Thinking of all the prescription pain relievers you have used **in the last 12 months**, if you could have had any of them and cost, availability, and access was not a problem, which would you have preferred? Please think of an answer in terms of using the drug in a way not directed by your healthcare provider.

| Xtampza® ER pill |
| --- |
| Oxycontin® pill |
| Hysingla® ER pill |
| Generic extended release single ingredient hydrocodone pill |
| Generic extended release pill containing only oxycodone |
| Immediate release pill containing only oxycodone |
| Another prescription pain reliever medication not listed here |

When you used a **<PRODUCT>** in the past in a way not directed by your healthcare provider, did you ever…

| …start taking more Xtampza® ER than your doctor recommended? |
| --- |
| …have to call an ambulance or go to the emergency room because you took too much Xtampza® ER? |
| …receive naloxone (Narcan®, Evzio®, or other generics) because you took too much Xtampza® ER? |
| …take Xtampza® ER with a prescription sedative (a medication used to treat insomnia, trouble sleeping, anxiety, panic disorders, or seizures)? |
| …drink alcohol while taking Xtampza® ER? |

In the past 12 months when you used a **<PRODUCT>** in a way not directed by your healthcare provider, did you…

| …swallow the pill whole? |
| --- |
| …open up or break apart the pill? |
| …chew the pill? |
| …crush the pill? |
| …heat or melt the pill? |
| …dissolve the pill in your mouth? |
| …dissolve the pill into a liquid? |
| …take the pill with another drug or medication? |

Please rate how difficult it was to **<METHOD>** your **<PRODUCT> pill**.

| Very easy |
| --- |
| Easy |
| Difficult |
| Very difficult |

What are all the reasons you **<METHOD>** your **<PRODUCT> pill** in the last 12 months?

| To swallow the pill more easily |
| --- |
| To inject the contents of the pill |
| To smoke or vape the contents of the pill |
| To snort the contents of the pill |
| To increase the high feeling from the pill |
| To improve the pain relief from the pill |
| To feel the effects of the pill more quickly |
| For another reason |

How much do people risk harming themselves physically and in other ways when they use **<PRODUCT**in a way not directed by a healthcare provider?

| No Risk |
| --- |
| Slight Risk |
| Moderate Risk |
| Great Risk |

How much do people risk harming themselves physically and in other ways when they use Oxycontin® in a way not directed by a healthcare provider?

| No Risk |
| --- |
| Slight Risk |
| Moderate Risk |
| Great Risk |

How much do people risk harming themselves physically and in other ways when they use Hysingla® ER in a way not directed by a healthcare provider?

| No Risk |
| --- |
| Slight Risk |
| Moderate Risk |
| Great Risk |

How much do people risk harming themselves physically and in other ways when they use a generic extended release tablet containing only hydrocodone in a way not directed by a healthcare provider?

| No Risk |
| --- |
| Slight Risk |
| Moderate Risk |
| Great Risk |

How much do people risk harming themselves physically and in other ways when they use a generic extended release pill containing only oxycodone in a way not directed by a healthcare provider?

| No Risk |
| --- |
| Slight Risk |
| Moderate Risk |
| Great Risk |

How much do people risk harming themselves physically and in other ways when they use an immediate release pill containing only oxycodone (Oxaydo®, Oxecta®, Roxicodone®, or generics) in a way not directed by a healthcare provider?

| No Risk |
| --- |
| Slight Risk |
| Moderate Risk |
| Great Risk |
