# Supplementary material for: Motivations, perceived risk, and tampering of XTAMPZA ER and abuse-deterrent opioid drugs
Source: PLoS One. 2025 Oct 8;20(10):e0332574. doi: 10.1371/journal.pone.0332574 (PMC12507222; doi:10.1371/journal.pone.0332574)
Supplement: Attachment — Submitted filename: PLOS ONE_XTAMPZA ER.docx [file pone.0332574.s004.docx]

The authors present an interesting study that warrants discussion. Given the ongoing opioid epidemic, the public health implications that studies of this nature explore are noteworthy. While the present study raises important questions regarding a public health crisis, I do have a few questions and suggestions.

There are multiple sentences that are confusing (e.g., sentence starting on line 73).

**Introduction**

- ER is clarified as “extended-release” in the first paragraph and does not need to be re-clarified again (e.g., start of the third paragraph).
- See previous comment for immediate release (IR).
- What is the nationally representative survey participants were recruited from?

**Methods**

- I am confused about what survey participants were pulled from. You mention RADARS and NMURx, but it is not clear how both surveys were utilized in this study.
- The first paragraph needs to be split into two.
- The description of each of the focal variables is difficult to discern since everything is mentioned in a single paragraph. There is no mention of the sociodemographic variables (mentioned in the results section).

**Results**

- I think there is a formatting issue with the sub-header lines for “Figure 1…” and for “Figure 2…”

**Discussion**

- You do not clarify what the abbreviation of FDA refers to, even though it is a commonly discussed agency in the US. You mention the agency’s full name earlier in the paper, but do not include the abbreviation.
