# Supplementary material for: Motivations, perceived risk, and tampering of XTAMPZA ER and abuse-deterrent opioid drugs
Source: PLoS One. 2025 Oct 8;20(10):e0332574. doi: 10.1371/journal.pone.0332574 (PMC12507222; doi:10.1371/journal.pone.0332574)
Supplement: Attachment — Submitted filename: Author Response.docx [file pone.0332574.s006.docx]

To the editors:

Thank you for your interest and the opportunity to revise our manuscript. We have responded to the reviewer comments below and uploaded a modified manuscript for consideration. We have also updated manuscript formatting based on the PLOS One style guide.

Editor Comment 1: Revise the objective to succinctly state the purpose and method of the study in more accessible terms.

AUTHOR RESPONSE EDITOR COMMENT 1: We have revised the objective and stated it plainly beginning on line 107.

Editor Comment 2: Provide a brief background on ADFs and their purpose before moving to specific examples like XTAMPZA ER.

AUTHOR RESPONSE EDITOR COMMENT 2: We added a sentence describing that ADF’s are an intervention to reduce the risks of ER opioid misuse by creating physical barriers to tampering beginning on line 56.

Editor Comment 3: While the DAST-10 threshold for substance use disorder is mentioned, include a brief justification for using this specific tool and how it contributes to the study outcomes.

AUTHOR RESPONSE EDITOR COMMENT 3: We added description of why we characterized the sample in terms of problematic drug use behavior beginning on line 110 and a description of the DAST-10 validated instrument beginning on line 153.

Editor Comment 4: Some sentences are very long and packed with multiple pieces of information. Break them into shorter, clearer sentences to improve readability.

AUTHOR RESPONSE EDITOR COMMENT 4: We edited the full manuscript and broke up sentences and paragraphs where necessary to improve readability.

Editor Comment 5: Consider defining terms like "RADARS System" and "DAST-10" for readers who may not be familiar with these methodologies.

AUTHOR RESPONSE EDITOR COMMENT 5: We removed mention of the RADARS System and added a description of the DAST-10 beginning on line 153. We reviewed the manuscript for additional terms which were possibly not well defined and added more complete definitions.

Editor Comment 6: In the discussion section, the ideas are densely packed and occasionally jump between topics (e.g., tampering behaviors, prescribing practices, and study strengths). Consider organizing the discussion into distinct paragraphs for each main topic (e.g., prevalence of NMU, tampering motivations, prescribing practices, study strengths, and limitations).

AUTHOR RESPONSE EDITOR COMMENT 6: We have reorganized the discussion section by main topics, ensuring multiple topics do not appear within the same paragraph.

Editor Comment 7: Expand on the implications of using real-world evidence (RWE) in post-market surveillance and how this study aligns with FDA guidance. Consider tying this to broader trends in RWE uses for regulatory decision-making

AUTHOR RESPONSE EDITOR COMMENT 7: We added a deeper discussion of the need for real-world evidence in post-marketing surveillance as well as a description of how our study meets those needs beginning on line 267.

**Reviewer #1**

Comment 1: The authors conducted an online survey using the RADARS System. However, I think this survey could not achieve the authors' aim because of the issue of the online panel. The respondents of this survey are limited to non-medical use opioid survivors. And vice versa, people who had died or experienced severe harm by the non-medical use of opioids could not answer this survey. Thus, I think that this study could not show the actual effect of ADF on non-medical use of opioids.

AUTHOR RESPONSE 1: Survivor bias is a limitation of this study as it would be with any data source that uses retrospective self-reported individual level data. I have added this as a limitation to the current study beginning on line 320.

Comment 2: Moreover, the result that only 628 of 9158 respondents answered the follow-up survey suggests that the respondents might be biased toward those with high literacy. People who do not have high literacy may use and tamper more than respondents.

AUTHOR RESPONSE 2: Of the 9,158 respondents who were invited to initiate the follow-up survey, 2,186 initiated the follow up survey, but only 738 were eligible to complete the survey based on past year reported NMU. This information is clarified beginning on line 189.

Comment 3: In the results section, the authors indicated that responders can endorse more than one drug. In other words, each drug group includes concomitant use of other formula opioids. Therefore, I assume that these results could not show the actual number of regarding characteristics, tampering, and other items in only XTAMPZA ER use.

AUTHOR RESPONSE 3: Respondents who endorsed more than one product were asked all relevant follow up questions separately in regards to each product. All reported metrics are specific to the products in each drug group presented.

Comment 4: I am also wondering why the authors did not consider the consumption of XTAMPZA ER and the opioids used as a comparator in this study.

AUTHOR RESPONSE 4: Consumption of XTAMPZA ER by several routes was considered and described in Table 2.

Comment 5: For these reasons, I'm afraid I have to disagree with the author's conclusion: "Among similar drugs, XTAMPZA ER was not the drug of choice for non-medical use, and popular methods of tampering may not involve increased risk of harmful outcomes."

AUTHOR RESPONSE 5: Among adults that reported NMU of a qualifying product, less than 3% reported XTAMPZA ER as their preferred drug. We have added clarification in the results and discussion sections that all conclusions are specific to the sample of adults reporting NMU.

Comment 6: To better address the research objectives, I suggest that authors consider combining the results of several surveys derived from different data sources in future studies. Consider Mortality Records, Emergency Department Visit and Hospitalization data, Poison Center data, etc.

AUTHOR RESPONSE 6: The objective of this work is to characterize how adults who report NMU of ADF products and their comparators use and tamper with these products in a community setting. This requires self-reported data sampled from the general population to capture adults who do not interact with the healthcare system. The goal of this work is to compliment the clinical studies that have been conducted. Such a study using clinical data has already been published by our group: <https://doi.org/10.1093/pm/pnaa272>. This work expands upon the past work by adding information from those in a community setting.

Comment 7: While the study addresses an important issue, the limitations in the methodology significantly constrain its ability to answer the research question. For these reasons, I recommend rejecting the manuscript in its current form. However, the study topic remains important, and I encourage the authors to consider revising their approach to better align with their research objectives.

AUTHOR RESPONSE 7: The research objective has been more clearly stated beginning on line 101. The manuscript has been reframed to more directly meet those objectives.

**Reviewer #2**

Comment 1: There are multiple sentences that are confusing (e.g., sentence starting on line 73).

AUTHOR RESPONSE 1: We have edited the full manuscript and updated any sentences that were confusing.

Comment 2: ER is clarified as “extended-release” in the first paragraph and does not need to be re-clarified again (e.g., start of the third paragraph).

AUTHOR RESPONSE 2: We removed the clarification.

Comment 3: See previous comment for immediate release (IR).

AUTHOR RESPONSE 3: We removed the clarification.

Comment 4: What is the nationally representative survey participants were recruited from?

AUTHOR RESPONSE 4: The nationally representative survey is the Survey of Non-Medical Use of Prescription Drugs (NMURx) routine survey. We added this clarification beginning on line 117.

Comment 5: I am confused about what survey participants were pulled from. You mention RADARS and NMURx, but it is not clear how both surveys were utilized in this study.

AUTHOR RESPONSE 5: Survey participants for the follow-up survey were recruited from the NMURx routine survey which is launched 2 times annually as part of the RADARS system. We have removed reference to RADARS as it is not directly relevant and reworked the methods section to clearly describe the two NMURx surveys and how they were each used for this research. Past validation studies for the NMURx survey are cited.

Comment 6: The first paragraph [of the methods] needs to be split into two.

AUTHOR RESPONSE 6: We split the first paragraph of the methods into one paragraph describing the routine NMURx survey and a second paragraph describing the follow-up survey.

Comment 7: The description of each of the focal variables is difficult to discern since everything is mentioned in a single paragraph.

AUTHOR RESPONSE 7: We have reorganized the methods section to describe each of the variables one at a time split thoughtfully into 3 paragraphs.

Comment 8: There is no mention of the sociodemographic variables (mentioned in the results section).

AUTHOR RESPONSE 8: We added a description of the sociodemographic variables taken from the routine NMURx survey beginning on line 136.

Comment 9: I think there is a formatting issue with the sub-header lines for “Figure 1…” and for “Figure 2…”

AUTHOR RESPONSE 9: We have updated the sub-headers to both figures and addressed the formatting errors.

Comment 9: You do not clarify what the abbreviation of FDA refers to, even though it is a commonly discussed agency in the US. You mention the agency’s full name earlier in the paper, but do not include the abbreviation.

AUTHOR RESPONSE 9: We added a clarifying abbreviation following the first mention of the agency’s full name on line 91.
