# Supplementary material for: Motivations, perceived risk, and tampering of XTAMPZA ER and abuse-deterrent opioid drugs
Source: PLoS One. 2025 Oct 8;20(10):e0332574. doi: 10.1371/journal.pone.0332574 (PMC12507222; doi:10.1371/journal.pone.0332574)
Supplement: Attachment — Submitted filename: Author_Response_auresp_2.docx [file pone.0332574.s007.docx]

To the editors:

Thank you for your interest and the opportunity to revise our manuscript. We have responded to the reviewer comments below and uploaded a modified manuscript for consideration. We have also updated manuscript formatting based on the PLOS One style guide.

**Reviewer #1**

Comment 1: Line 18: I could not understand the meaning of the term "drug use profiles" until I read the Discussion(line 227). I recommend that the authors add some explanation.

AUTHOR RESPONSE 1: We added an explanation for this term in the abstract beginning on line 18.

Comment 2: Line 20: I guess 16％ is "non-"ADF ER.

AUTHOR RESPONSE 2: This has been updated to be more clear.

Comment 3: Are decimal points in percentages rounded down? Is it rounded off? It does not seem to be consistent.

AUTHOR RESPONSE 3: All percentages have been rounded to 1 decimal place.

**Materials and Methods**

Comment 4: Even if the authors wrote about the sponsor of this research in Acknowledgment, I think that it should also be written in Materials and Methods.

AUTHOR RESPONSE 4: Funding disclosure added, beginning on line 137.

Comment 5: Line 106: I understand the classification of comparable products, however, I could not know how the authors chose comparable products. Additionally, the list of products should be shown in a table.

AUTHOR RESPONSE 5: Comparable products were selected based on having similar indication and forms to XTAMPZA ER. This has been clarified beginning on line 122 and a full list of products included in each group has been added as an appendix table.

Comment 6: Lines 30-43: The authors wrote the number of participants. It should also be shown as a flow diagram.

AUTHOR RESPONSE 6: Flow chart was added as figure 1 on line 163.

Comment 7: The questionnaire of this survey should be shown.

AUTHOR RESPONSE 7: Wording of the survey has been included as an appendix.

**Result**

Comment 8: Table 2: In the "Reason for Tampering", "To increase the high feeling from the pill", "To improve the pain relief from the pill", and "To feel the effects of the pill more quickly" should be separate from other items. I think that "To swallow", "inject", "smoke or vape", and "snort" mean only change the route, do not mean the reasons. These are different concepts.

AUTHOR RESPONSE 8: The reasons described in the manuscript reflect the way the questions were asked on the survey (exact survey language added as supplemental). Because these products are not intended for non-oral use, use by other routes motivates tampering with the pills and that motivation was considered a reason in how the question was developed. This reasoning was added beginning on line 133.

Comment 9: Table 2: In the "Risk Behaviors and Outcomes" should be distinguished from "Risk Behaviors" and "Outcomes".

AUTHOR RESPONSE 9: This table has been updated to separate outcomes from risk behaviors.

Comment 10: Figures should be improved. I can imagine what the authors want to show in the Figures, however, it is unclear on what basis each point is placed in its respective position. I know the two X axes show "mean difficulty" and "mean perceived risk of tampering", respectively. The actual value of the respective means should also be specified somewhere.

AUTHOR RESPONSE 10: Actual means corresponding to each point have been added as captions to both figures. The methods have also been updated to include the numerical values of the Likert scale for better interpretability of the means.

**Discussion**

Comment 11: Lines 214: The figures 33.8％ and 7.7% are not shown in the Result section and Tables. It is unfriendly for readers. I recommend that the authors show these in the Results section.

AUTHOR RESPONSE 11:

Comment 12: The number of consumptions, the number of prescriptions dispensed, or the ratio of the products surveyed in this study in the US is required. If XTAMPZA is not commonly prescribed and distributed in the US, the number of participants who non-medically use XTAMPZA will also be small.

AUTHOR RESPONSE 12: The proportion of dispensing for each comparator group relative to XTAMPZA ER was added beginning on line 154.

**Reviewer #2**

Comment 1: Please double-check punctions and grammar.

AUTHOR RESPONSE 1: Both coauthors and myself have reviewed the manuscript for punctuation and grammar, making several corrections.

**Introduction**

Comment 2: There is an extra “.” On line 41.

AUTHOR RESPONSE 2: Extra punctuation removed.

Comment 3: Need to capitalize the start of the sentence on line 74.

AUTHOR RESPONSE 3: “f” changed to “F”.

Comment 4: Missing a period at the end of the sentence on line 76.

AUTHOR RESPONSE 4: punctuation added.

Comment 5: There is an extra “.” and “,” on line 80.

AUTHOR RESPONSE 5: Extra punctuation removed

Comment 6: You explain what NMU stands for in line 95, but it needs to occur at the first usage of NMU (line 85).

AUTHOR RESPONSE 6: definition of NMU abbreviation added to first instance on line 86 and removed from line 96.

**Results**

Comment 7: This may be an oversight on my end, but I do not see where Figures 1 or 2 are supposed to be placed in the manuscript.

AUTHOR RESPONSE 7: Figure 1 has a title/caption beginning on line 196 and figure 2 has a title/caption beginning on line 212.

**Discussion**
Comment 8: The limitations (starts on line 260) should be a separate paragraph.

AUTHOR RESPONSE 8: The limitations have been split into a paragraph separate from the strengths, beginning on line 274, with some added content.

Comment 9: Your last paragraph is a single sentence. Expanding this paragraph with a couple more sentences would help highlight the novelty and implications of this study.

AUTHOR RESPONSE 9: The concluding paragraph has been expanding to highlight the novelty and important results of this study.
