# Supplementary material for: Motivations, perceived risk, and tampering of XTAMPZA ER and abuse-deterrent opioid drugs
Source: PLoS One. 2025 Oct 8;20(10):e0332574. doi: 10.1371/journal.pone.0332574 (PMC12507222; doi:10.1371/journal.pone.0332574)
Supplement: Attachment — Submitted filename: Author_Response_auresp_3.docx [file pone.0332574.s008.docx]

To the editors:

Thank you for your interest and the opportunity to revise our manuscript. We have responded to the reviewer comments below and uploaded a modified manuscript for consideration. We have also updated manuscript formatting based on the PLOS One style guide.

**Reviewer #3**

Comment 1: Reviewer #3: Based on the revised manuscript and the authors' responses to reviewers, the paper is well-structured, data-rich, and addresses major reviewer concerns. However, a few key areas for further improvement remain that could enhance the manuscript’s clarity, rigor, and reader trust. Some part of the abstract and discussion are still wordy. This may hinder understanding for broader audiences. Perform a final language polish focusing on removing redundant phrases like in line 285-286. Simplifying overly long sentences, in the Abstract (line 18–19) and Discussion (line 287- 289).

AUTHOR RESPONSE 1: We performed a thorough round of editing, focusing on removing redundant text and simplifying long sentences.
